# Supplementary material for: V2C-Long: Longitudinal cortex reconstruction with spatiotemporal correspondence
Source: Imaging Neurosci (Camb). 2025 Mar 7;3:imag_a_00500. doi: 10.1162/imag_a_00500 (PMC12319792; doi:10.1162/imag_a_00500)
Supplement: Supplementary Material [file imag_a_00500-supp.pdf]

# V2C-Long — Supplementary Material

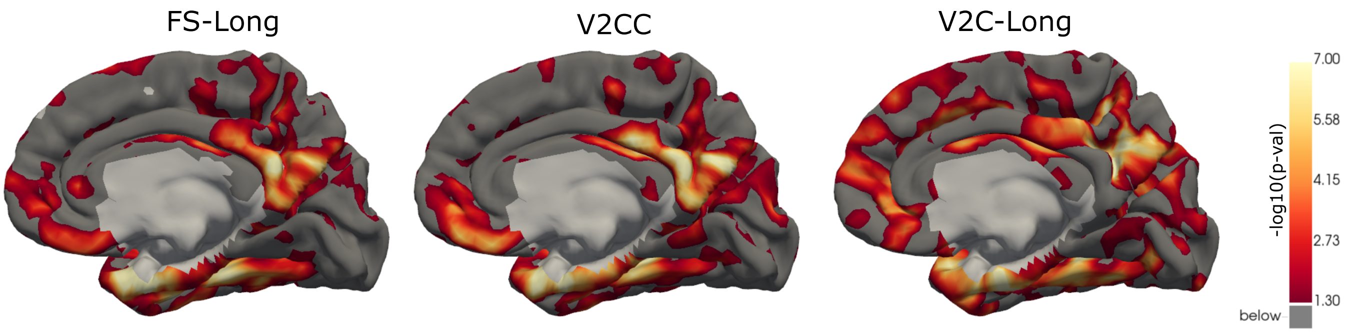


Supplementary Figure 1: Medial view on differences in longitudinal cortical thickness between stable AD subjects ($n = 71$) and healthy controls ($n = 193$). We show uncorrected negative log10(p-value)-maps on FsAverage — based on meshes from FS-Long (v7.2), V2CC, and V2C-Long.

Supplementary Table 1: Results from a joint V2C-Long model trained for the left (lh) and right (rh) hemisphere. We report accuracy (ASSD) and consistency (ParcF1) metrics by surface and hemisphere. Values are mean±SD over all subjects (ParcF1) respectively all scans (ASSD) in our ADNI test set.

| WM surface lh | | Pial surface lh | | WM surface rh | | Pial surface rh | |
|---|---|---|---|---|---|---|---|
| ASSD↓ | ParcF1↑ | ASSD↓ | ParcF1↑ | ASSD↓ | ParcF1↑ | ASSD↓ | ParcF1↑ |
| 0.170±0.055 | 0.967±0.013 | 0.163±0.059 | 0.962±0.015 | 0.169± 0.053 | 0.969±0.011 | 0.162±0.060 | 0.963±0.014 |

Supplementary Table 2: Destrieux atlas regions in counter-clockwise order.

G_insular_short
G_occipital_middle
G_occipital_sup
G_oc-temp_lat-fusifor
G_oc-temp_med-Lingual
G_oc-temp_med-Parahip
G_orbital
G_pariet_inf-Angular
G_pariet_inf-Supramar
G_parietal_sup
G_postcentral
G_precentral
G_precuneus

Supplementary Table 2: Destrieux atlas regions in counter-clockwise order (continued).

| |
|---|
| G_rectus |
| G_subcallosal |
| G_temp_sup-G_T_transv |
| G_temp_sup-Lateral |
| G_temp_sup-Plan_polar |
| G_temp_sup-Plan_tempo |
| G_temporal_inf |
| G_temporal_middle |
| Lat_Fis-ant-Horizont |
| Lat_Fis-ant-Vertical |
| Lat_Fis-post |
| Pole_occipital |
| Pole_temporal |
| S_calcarine |
| S_central |
| S_cingul-Marginalis |
| S_circular_insula_ant |
| S_circular_insula_inf |
| S_circular_insula_sup |
| S_collat_transv_ant |
| S_collat_transv_post |
| S_front_inf |
| S_front_middle |
| S_front_sup |
| S_interm_prim-Jensen |
| S_intrapariet_and_P_trans |
| S_oc_middle_and_Lunatus |
| S_oc_sup_and_transversal |
| S_occipital_ant |
| S_oc-temp_lat |
| S_oc-temp_med_and_Lingual |
| S_orbital_lateral |
| S_orbital_med-olfact |

Supplementary Table 2: Destrieux atlas regions in counter-clockwise order (continued).

S_orbital-H_Shaped
S_parieto_occipital
S_pericallosal
S_postcentral
S_precentral-inf-part
S_precentral-sup-part
S_suborbital
S_subparietal
S_temporal_inf
S_temporal_sup
S_temporal_transverse
Unknown
G_and_S_frontomargin
G_and_S_occipital_inf
G_and_S_paracentral
G_and_S_subcentral
G_and_S_transv_frontopol
G_and_S_cingul-Ant
G_and_S_cingul-Mid-Ant
G_and_S_cingul-Mid-Post
G_cingul-Post-dorsal
G_cingul-Post-ventral
G_cuneus
G_front_inf-Opercular
G_front_inf-Orbital
G_front_inf-Triangul
G_front_middle
G_front_sup
G_Ins_lg_and_S_cent_ins

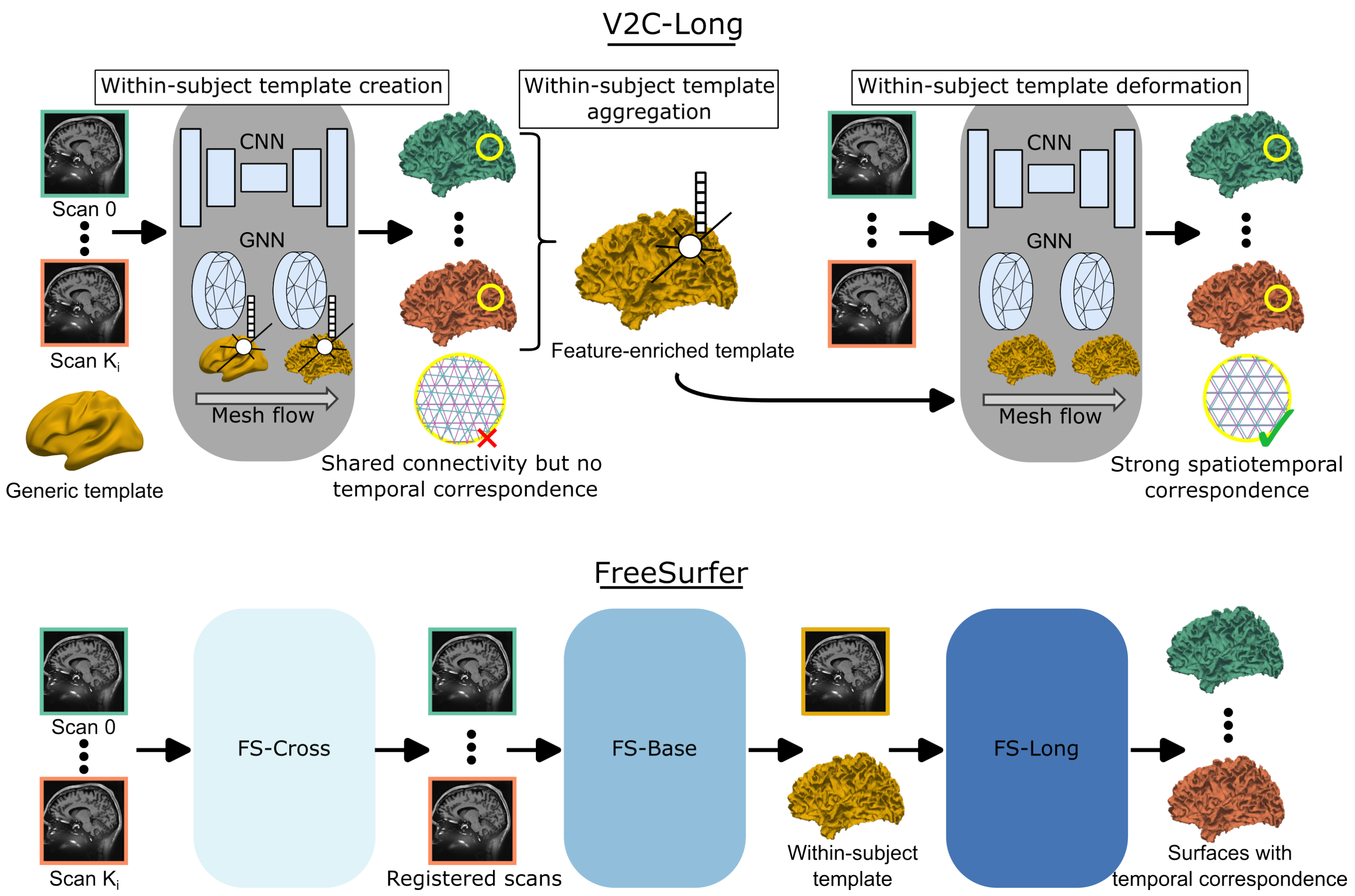


Supplementary Figure 2: Conceptual comparison of V2C-Long with the longitudinal FreeSurfer pipeline.

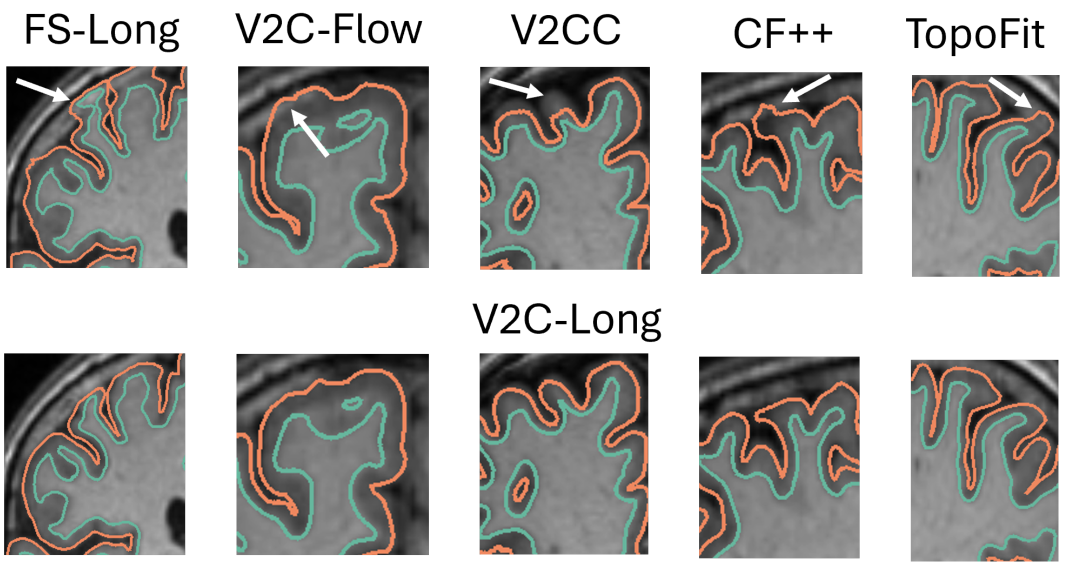


Supplementary Figure 3: Failure cases of existing methods (indicated by white arrows, top row) and corresponding V2C-Long segmentation (bottom row).

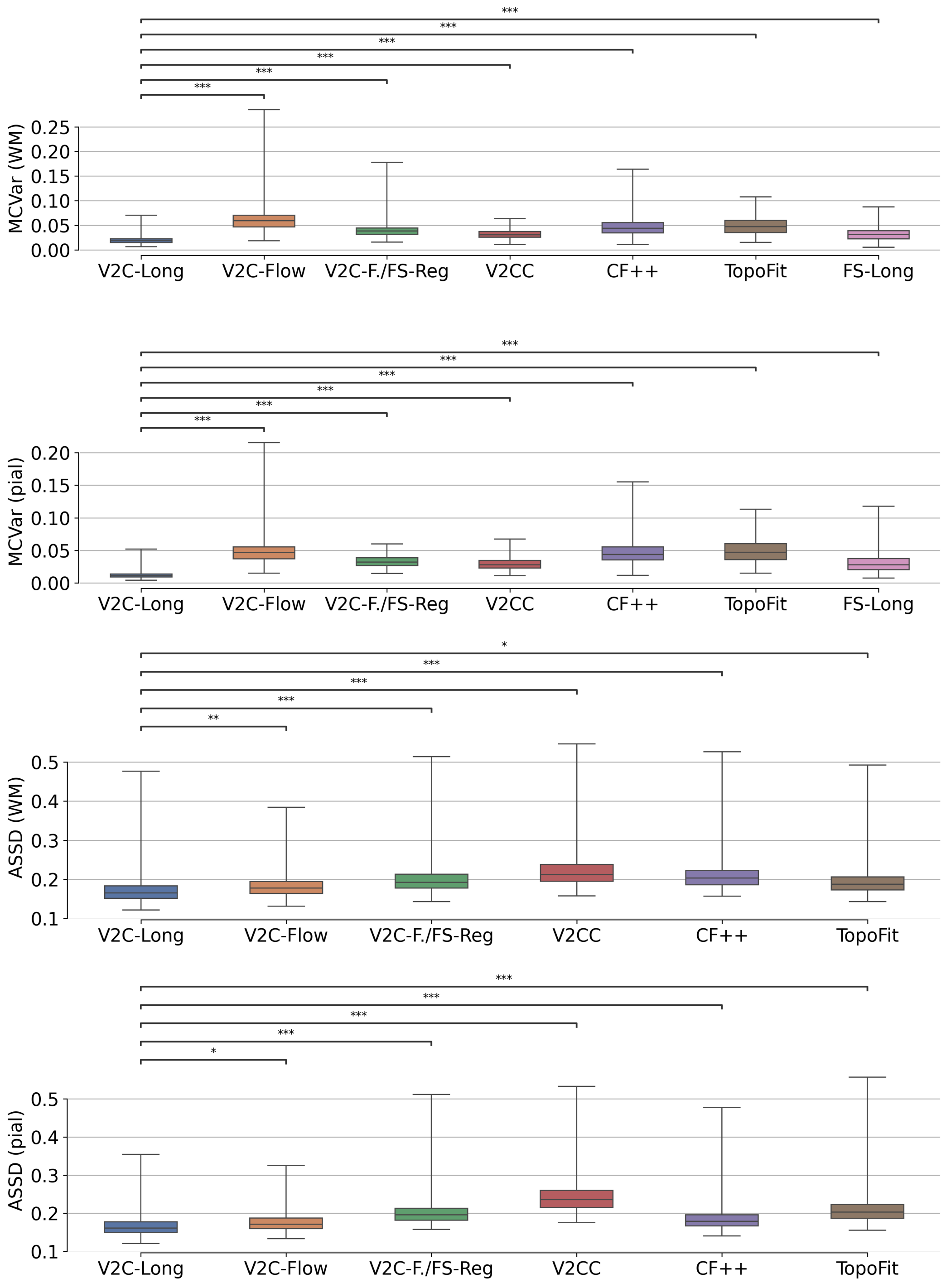


Supplementary Figure 4: Longitudinal consistency (MCVar) and reconstruction accuracy (ASSD) for white matter (WM) and pial surfaces in our ADNI test set. Boxplots show the median as the central line, boxes representing the quartiles, and whiskers extending to 10 times the interquartile range. Stars indicate significance levels based on paired t-tests between the scores of V2C-Long and the other methods. $^{***}: p < 1\text{e}{-10}$, $^{**}: p < 1\text{e}{-5}$, $^{*}: p < 0.01$.

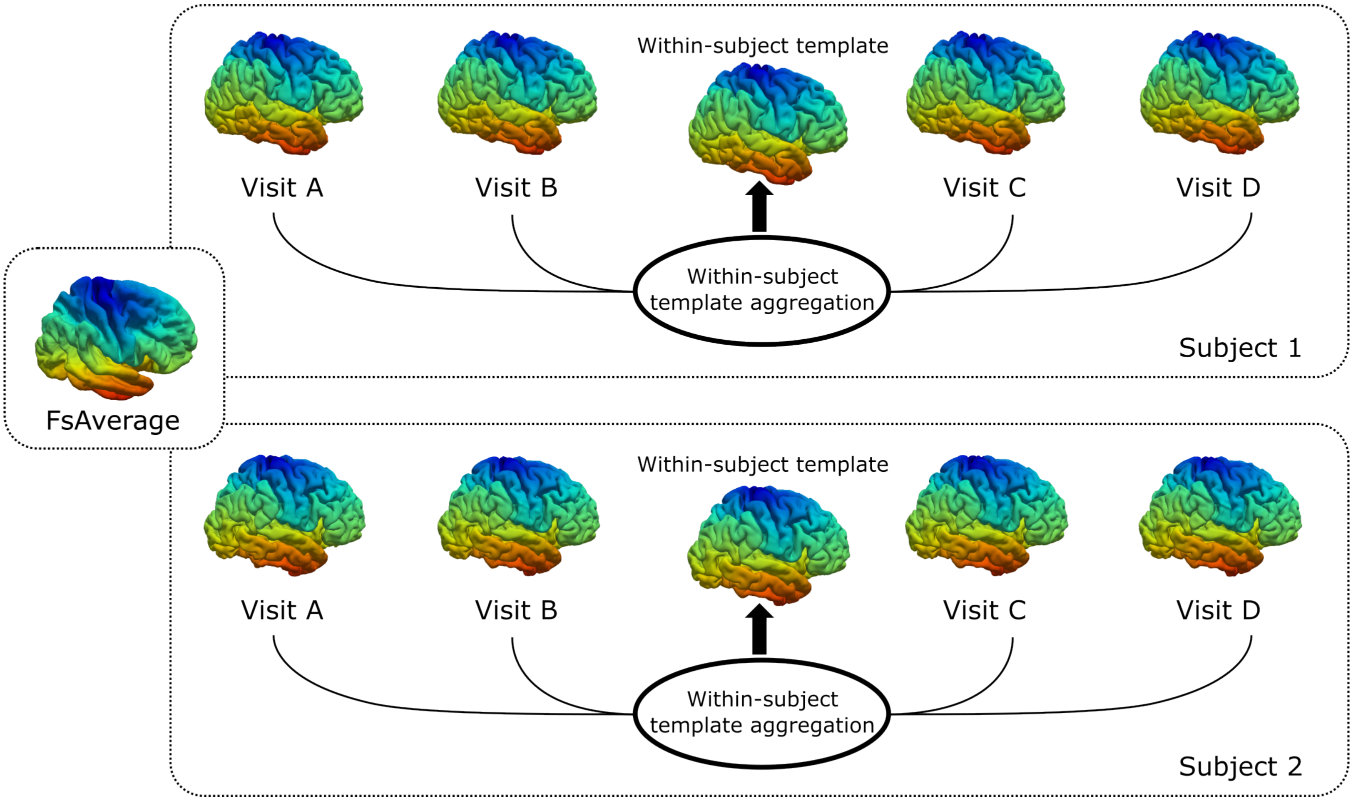


Supplementary Figure 5: Visualization of the correspondences to FsAverage that permit inter- and intra-subject averaging of vertices. We show pial surfaces, reconstructed with V2C-Flow, from two subjects in our ADNI test set and four visits each, together with the obtained within-subject template in V2C-Long. Every vertex is assigned an individual color so that correspondences can be identified by matching colors.

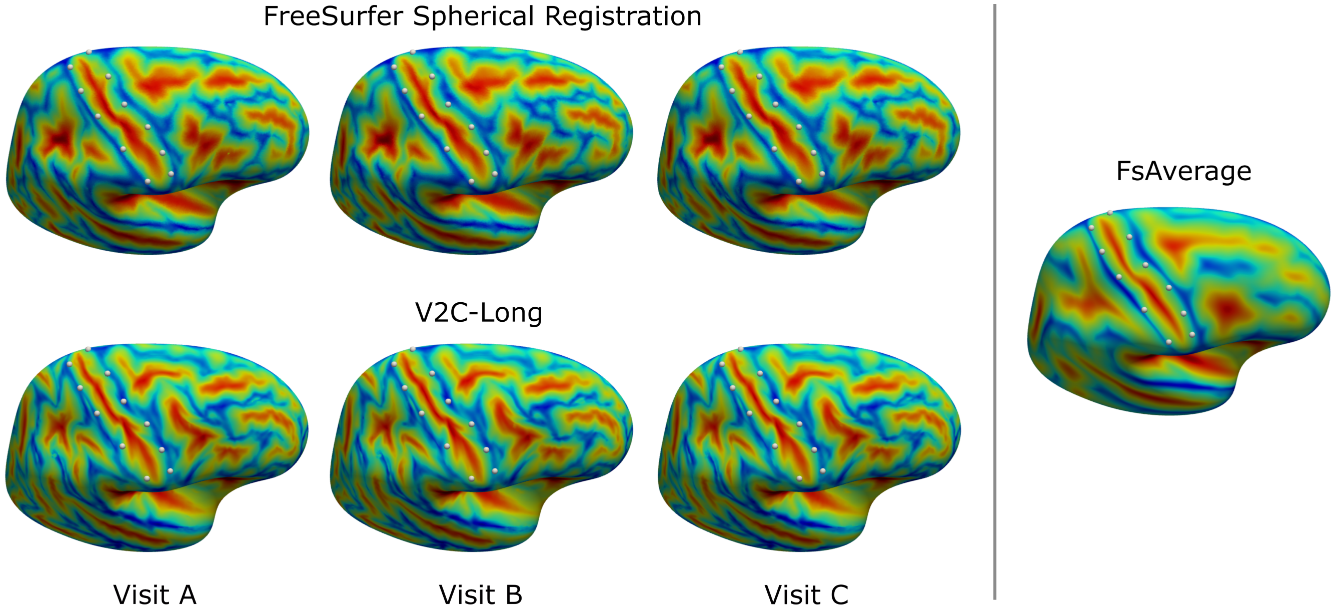


Supplementary Figure 6: Visualization of the within-subject correspondences and the correspondences to FsAverage based on sulcal depth maps. Shown is the inflated FsAverage mesh, with vertices around the central sulcus marked in white. After FreeSurfer's spherical registration, values were resampled to the FsAverage mesh; values from V2C-Long were mapped directly based on the inherent vertex correspondence. Ideally, sulcal depth values match at a certain vertex. The scans used for this analysis originate from our ADNI test set.
